# Role of apolipoprotein E epsilon 4 (APOE*ε4) as an independent risk factor for incident depression over a 12-year period in cognitively intact adults across the lifespan

**DOI:** 10.1192/bjo.2020.29

**Published:** 2020-05-08

**Authors:** Richard Andrew Burns, Shea Andrews, Nicolas Cherbuin, Kaarin Jane Anstey

**Affiliations:** Centre for Research on Ageing, Health and Wellbeing, The Australian National University, Australia; Department of Neuroscience, Icahn School of Medicine at Mount Sinai, USA; Centre for Research on Ageing, Health and Wellbeing, The Australian National University, Australia; School of Psychology, University of New South Wales, Australia; and Neuroscience Research Australia, Australia

**Keywords:** Apolipoprotein, depression, cognitive ageing, dementia, lifespan, epidemiology

## Abstract

**Background:**

The apolipoprotein E ε4 allele (APOE*ε4) is indicated as a risk for Alzheimer's disease and other age-related diseases. The risk attributable to APOE*ε4 for depression is less clear and may be because of confounding of the relationship between dementia and depression.

**Aims:**

We examined the risk of APOE* ε4 for incident depression and depressive symptomology over a 12-year period across the adult lifespan.

**Method:**

Participants were from the Personality and Total Health Through Life study, aged 20 to 24 (*n* = 1420), 40 to 44 (*n* = 1592) or 60–64 (*n* = 1768) at baseline, and interviewed every 4 years since 1999. Ethnicities other than White, those without genotyping and those with depression at baseline, or who reported strokes and scores on the Mini-Mental State Examination <27 at any observation, were excluded.

**Results:**

Over the study period, there was no evidence that APOE*ε4*+* was a risk factor for depression, including any depression (odds ratio (OR) = 0.94, 95% CI 0.77–1.16, *P* = 0.573), major depression (OR = 0.96, 95% CI 0.60–1.53, *P* = 0.860), minor depression (OR = 0.94, 95% CI 0.67–1.30, *P* = 0.695) or depressive symptomology (incidence rate ratio (IRR) = 1.02, 95% CI 0.97–1.08, *P* = 0.451). APOE*ε4 was unrelated to incident depression. Findings were consistent for all age cohorts.

**Conclusions:**

Among cognitively intact Australian adults who were free of depression at baseline, there was little evidence that APOE*ε4*+* carriers are at increased risk for depression over a 12-year period among those who are cognitively intact.

## Apolipoprotein E, dementia and age-related morbidity

The risk of apolipoprotein E ε4 allele (APOE*ε4) for dementia is well established.^1–4^ An antagonistic pleotropic hypothesis posits that the risk attributed to APOE*ε4 is evident in late life; in childhood and early adulthood APOE*ε4 confers a cognitive benefit.^5^ Evidence for this benefit in mid-adulthood is mixed; studies that combine young and middle age continue to report benefit,^6,7^ but one study that focused exclusively on a mid-life cohort identified no benefit, but also no risk for decrement in cognitive function related to APOE*ε4 status.^8^ APOE*ε4 is also indicated as a pleiotropic factor for other age-related diseases including atherosclerosis, cardiovascular and cerebrovascular diseases.^2,9,10^

## APOE*ε4 and depression

There is also some debate about the role of APOE*ε4 in depression. Evidence is mixed with support for and against the risk attributed to APOE*ε4 in the aetiology of depression.^11–18^ Conclusions are typically drawn from clinical studies with comparatively small sample sizes and that typically comprise vulnerable or at-risk populations, or larger population-based studies that are often cross-sectional or retrospective in design. Given the known risk of APOE* ε4 for dementia,^1–3^ elucidation of the APOE*ε4–depression link is further confounded by dementia pathology that may be precursors to neurocognitive disorders.^16,19^ Further confounds include the association between depression and dementia.^20–22^ However, recently, APOE*ε4 was not indicated in a genome-wide analysis of depression with 807 553 individuals.^23^ One major systematic review and meta-analysis indicated that APOE*ε4 was a risk for late-life depression only, but only in contrast to those with the APOE*ε3 alleles, and this effect was driven by a single and very small clinical study.^24^

There is therefore a need for large longitudinal population studies to examine the long-term prospective risk of APOE*ε4 for depression in which confounding of concurrent cognitive impairment and initial mental health are controlled. Most recently, the prospective APOE*ε4 4-year risk for increased depressive symptomology and incident depression status was reported in a Swedish study of 800 older adults who were depression-free at baseline and who remained free of significant cognitive decline over the study period.^17^ However, this contrasts with other longitudinal studies that identify no association between APOE*ε4 and depression.^25^ There remains a need to replicate these findings and to extend the examination of risk associated with APOE*ε4 over a longer follow-up, and to examine this risk across the lifespan. Longitudinal studies in which participants are repeatedly interviewed over several years allow us to examine long-term risk with multiple observations. To date, many studies are limited to annual to 4- or 5-year risk.^14,17,18^ We report here the 4-, 8- and 12-year risk of APOE*ε4 for incident depression and hypothesise that APOE*ε4 is a risk for depression, particularly for older adults.

## Method

### Participants

Participants were from the Personality and Total Health (PATH) Through Life project,^26^ a large community survey that was designed to chart the progression of mental health, cognitive function and substance use across adulthood and identify the individual characteristics and the environmental and genetic risk factors for health outcomes. Participants were randomly selected from the electoral rolls – voting is compulsory in Australia – of Canberra and Queanbeyan, Australia. A random selection of adults in this region who were aged in one of three age bands (20–24; 40–44; 60–64) were invited to participate. Response rates for the invitation were 58.6% for those aged 20 to 24 (*n* = 2404), 64.6% for those aged 40 to 44 (*n* = 2530), and 58.3% for those aged 60 to 64 (*n* = 2551) at baseline. Participants have been interviewed every 4 years since 1999/2000 with an average retention rate of 63% of the baseline sample by the fourth wave.

Results of the current paper presented here concern the first four waves of data collection for participants (*n* = 4780) who met our inclusion criteria for this study and provided buccal swabs for genotyping and necessary follow-up information on depression at 4-, 8- and 12-year follow-up. In the current paper, we excluded ethnicities other than White (*n* = 332) and individuals who had depression at baseline (*n* = 1213) or who reported stroke at any observation (*n_ob_*_s_ = 243). As a result of confounding between cognitive function, dementia, depression and APOE*ε4, we retained only cognitively intact participants excluding observations at each wave from participants in the 60s cohort (*n_obs_* = 317) who reported <27 on the Mini-Mental State Examination.^27^ Therefore sample size by age group was *n* = 1420 for those aged 20 to 24, *n* = 1592 for those aged 40 to 44 and *n* = 1768 for those aged 60 to 64 at baseline.

Participants were assessed in their own homes under the supervision of a professional interviewer. For wave 4, participants in the 20s and 40s cohort completed the survey questionnaire online prior to face-to-face cognitive, physical and clinical assessment whereas those in the 60s cohort did the questionnaire face-to-face. Participants received a full description of the study and provided informed consent. All procedures contributing to this work comply with the ethical standards of the relevant national and institutional committees on human experimentation and with the Helsinki Declaration of 1975, as revised in 2008. The study was approved by the Human Research Ethics Committee at the Australian National University.

### Measures

#### Depression

Depression was operationalised in terms of symptoms of depression and likely depression diagnosis. Mental health symptoms were assessed with the Goldberg Depression Scale (GDS).^28^ The GDS comprises a list of nine depression symptoms. Participants respond ‘yes’ or ‘no’ to whether they have experienced any of the symptoms. A total symptom count variable was created with a low score of zero reflecting no-symptoms reported, to nine, all symptoms endorsed. The scale reports high sensitivity to DSM diagnosis of depression.^28,29^ Minor and major depression diagnosis was made with the nine-item Brief Patient Health Questionnaire (BPHQ).^30–32^ In PATH, the GDS (area under the curve (AUC) = 0.88, 95% CI 0.84–0.92) and the BPHQ (AUC = 0.88, (95% CI 0.84–0.93) have excellent sensitivity and specificity against 30-day diagnoses of any depressive disorder as assessed with the Composite International Diagnostic Interview.^29^ As the BPHQ was not assessed for all age cohorts at baseline, we determined likely depression status according to a score of 5 or more on the GDS, which has established specificity and sensitivity for depression, including in PATH.^28,29^

#### Covariates

Analyses were adjusted for several variables including gender (reference: female), years of education and the physical health component scale from the Short-Form Health Survey-12.^33^

### *APOE* genotyping

Genotyping of the PATH sample has been previously described.^34^ Briefly, genomic DNA was extracted from buccal swabs using Qiagen Blood kits. Two TaqMan assays were performed to ascertain the genotypes of the two single nucleotide polymorphisms defining the *APOE* alleles, rs429358 and rs7412. Overall, 95.3% of the 20s cohort, 90.6% of the 40s cohort, and 90.1% of the 60s cohort provided buccal swabs. *APOE* genotype frequencies for the current study are presented in Table 1. Genotype frequencies did not deviate from Hardy–Weinberg equilibrium (20s cohort: χ^2^ = 3.37, d.f. = 3, *P =* 0.337; 40s cohort: χ^2^ = 0.72, d.f. = 3, *p =* 0.867; 60s cohort: χ^2^ = 2.92, d.f. = 3, *P* = 0.404). Participants with the *APOE* ε2/ε4 allele were excluded from the analysis to avoid conflation between the APOE*ε2 protective effects and *APOE ε4* risk effects.^35^ *APOE* alleles were coded as APOE*ε4+ (ε3/ε4 + ε4/ε4) or APOE*ε4− (ε2/ε2 + ε2/ε3 + ε3/ε3). Sensitivity analyses included models re-estimated with the binary APOE*ε4 carrier variable replaced by a count of the number of *ε4* alleles carriers possessed.

**Table 1:** Apolipoprotein E (*APOE*) genotype frequencies and characteristics of the PATH Study by age cohort^a^

	20s cohort (*n* = 2404)	40s cohort (*n* = 2530)	60s cohort (*n* = 2551)	Test statistics
Gender, *n* (%)				
Men	1162 (48.3)	1193 (47.2)	1317 (51.6)	χ^2^ = 10.91; *P* = 0.004
Education				
Years of education,^b^ mean (s.d.)	15.4 (1.8)	15.0 (2.3)	14.0 (2.7)	*F* = 247.71; *P* < 0.001
Physical health				
SF-12 PHC Score, mean (s.d.)	53.04 (6.84)	51.71 (7.99)	48.14 (10.13)	*F* = 209.71; *P* < 0.001
*Mental health*				
GDS, mean (s.d.)	2.9 (2.4)	2.4 (2.4)	1.7 (1.9)	*F* = 188.36; *P* < 0.001
BPHQ Depression,^c^ *n* (%)				
Minor depression	255 (4.8)	211 (3.4)	311 (3.8)	χ^2^ = 24.96; *P* < 0.001
Major depression	287 (5.4)	257 (4.1)	134 (1.6)	χ^2^ = 172.64; *P* < 0.001
Any depression	1128 (21.2)	981 (15.6)	937 (11.3)	χ^2^ = 24.32; *P* < 0.001
APOE genotype, *n* (%)				
ε2/ε2	14 (0.6)	15 (0.7)	19 (0.8)	
ε3/ε3	1411 (61.6)	1348 (58.7)	1444 (60.7)	
ε4/ε4	60 (2.6)	46 (2.0)	49 (2.1)	
ε2/ε3	248 (10.8)	298 (12.0)	274 (11.5)	
ε2/ε4	46 (2.0)	59 (2.6)	60 (2.5)	
ε3/ε4	512 (22.4)	532 (23.2)	532 (22.4)	
Any ε4	618 (26.0)	637 (27.7)	641 (26.0)	χ^2^ = 0.34; *P* = 0.844
Number of ε4 alleles, *n* (%)				
1 ε4 allele	512 (22.8)	532 (23.8)	532 (23.0)	χ^2^ = 13.41; *P* = 0.587
2 ε4 alleles	60 (2.7)	46 (2.1)	49 (2.1)	

SF-12 PHC, Short-Form 12 Physical Health component score; GDS, Goldberg Depression Scale; BPHQ, nine-item Brief Patient Health Questionnaire.

a. All variables reflect baseline characteristics except for education and BPHQ depression.

b. Owing to participants engaging with education across the life course we report highest years of education ever reported by a participant over the 12-year study period.

c. BPHQ depression reflect total number of incident depression over the 12-year study period.

### Statistical analysis

For comparison of baseline characteristics, age-group differences in proportions were tested with Pearson chi-square test. Differences in continuous variables were tested with a one-way ANOVA. *Post hoc* comparison between levels were undertaken with *t*-statistics and odds ratios (ORs) reported with Bonferroni correction. For the main analytical questions, analyses of depression symptomology were estimated with a Poisson regression, results of which were interpreted in terms of the incidence rate ratio (IRR). Analyses of depression diagnosis were estimated with a logistic regression; results were interpreted in terms of the OR. Longitudinal models were estimated within a multilevel framework which adjusts for the non-independence of repeated observations within individuals. Robust or Huber-White Sandwich standard errors were obtained and provide *P*-values corrected for heteroscedasticity.

Two main analytical approaches were undertaken. First, analysis examined the APOE*ε4 risk for depression over the whole study period, estimating individuals’ risk for incident depression and depressive symptomology over the 12 years in a multilevel framework. Second, individual estimates for the 4-, 8- and 12-year APOE*ε4 risk for depression were estimated. Analysis of the whole sample was first undertaken and then repeated stratified by age cohort. We examined possible modulation pathways by which APOE*ε4 risk for depression may develop by examining interactions between APOE*ε4 carrier status and gender, years of education and physical health. Eligible participants in the analysis sample (*n* = 4780), as defined previously, provided complete information on the covariates. We moderate decisions regarding purported associations between constructs based on ‘statistical significance’,^36^ and interpret estimates by evaluating the magnitude of the effect size and then considering its significance value.

Owing to non-response at individual waves for non-responders, we examined the likelihood of different non-responses among APOE*ε4 carriers. APOE*ε4 status was unrelated to likelihood of drop-out at 4- (OR = 1.01, 95% CI 0.82–1.26, *P* = 0.660) 8- (OR = 0.95, 95% CI 0.80–1.12, *P* = 0.528) or 12-year follow-up (OR = 1.04, 95% CI 0.91–1.19, *P* = 0.550)

## Results

There were differences between age groups across all variables except APOE*ε4 (Table 1). The 60s cohort had slightly higher proportions of men in comparison with the 20s (OR = 1.14 (95% CI 1.02–1.28), *P* = 0.021) and 40s (OR = 1.20 (95% CI 1.07–1.34), *P* = 0.001). The 20s cohort reported higher education than both the 40s (*t* = 7.26; *P* < 0.001) and the 60s (*t* = 21.48, *P* < 0.001). The 40s cohort reported higher education than the 60s cohort (*t* = 14.01, *P* < 0.001). The 20s cohort also reported higher scores on the GDS than both the 40s (*t* = 6.89, *P* < 0.001) and the 60s (*t* = 19.93, *P* < 0.001) whereas the 40s cohort also reported higher GDS scores than the 60s cohort (*t* = 12.40, *P* < 0.001). There were age-group differences in physical health with the 20s cohort being healthier than the 40s (*t* = 4.34, *P* < 0.001) and 60s (*t* = 14.25, *P* < 0.001) cohorts, and the 40s cohort healthier than the 60s cohort (t = 9.85, *P* < 0.001).

Over the study period there were differences in depression outcome between age groups. Those in the 20s cohort were more likely to report depression in comparison with those in their 40s (any depression: OR = 1.45 (95% CI 1.32–1.60, *P* < 0.001); minor depression OR = 1.53 (95% CI 1.27–1.84), *P* < 0.001; major depression: OR = 1.41 (95% CI 1.19–1.68), *P* < 0.001) and 60s (any depression: OR = 2.11 (95% CI 1.92–2.32), *P* < .001); minor depression: OR = 1.44 (95% CI 1.21–1.70) *P* < 0.001; major depression: OR = 3.75 (95% CI 3.04–4.62), *P* < 0.001). Those in the 40s cohort were more likely to report depression in comparison with those in the 60s cohort (any depression: OR = 1.45 (95% CI 1.32–1.60), *P* < .001) and major depression: OR = 2.66 (95% CI 2.15–3.29), *P* < 0.001), but not for minor depression (OR = 0.94 (95% CI 0.79–1.12), *P* = 0.502). These age differences justify stratification of analysis by age cohort.

### APOE*ε4*+* risk for depression over the study period

Over the study period, there was no evidence that APOE*ε4*+* was a risk factor for depression, including any depression (OR = 0.94 (95% CI 0.77; 1.16), *P* = 0.573), major depression (OR = 0.96 (0.60; 1.53), *P* = 0.860), minor depression (OR = 0.94 (0.67; 1.30), *P* = 0.695) or depressive symptomology (IRR = 1.02 (95% CI 0.97; 1.08), *P* = 0.451). The lack of risk was mostly consistent for all three age cohorts (Table 2). There was evidence for one very small effect for the 60s cohort whereby APOE*ε4 was associated with an increase in symptomology at a rate 1.13 times larger than those without APOE*ε4 status. Despite the lack of evidence for depression risk overall, we still examined interactions between APOE*ε4 with gender, years of education and physical health for the whole sample and by age cohort; no substantive effects were reported.

**Table 2:** Relationship between apolipoprotein E ε4 allele (APOE*ε*4*)+ and incident depression over the study period

	GDS		Any depression		Major depression		Minor depression	
	IRR (95% CI)	*P*	OR (95% CI)	*P*	OR (95% CI)	*P*	OR (95% CI)	*P*
20s cohort								
APOE*ε*4*+	1.04 (0.94–1.16)	0.458	0.88 (0.63–1.23)	0.444	0.87 (0.37–2.03)	0.744	0.90 (0.55–1.46)	0.662
40s cohort								
APOE*ε*4*+	0.92 (0.82–1.04)	0.172	0.73 (0.50–1.06)	0.101	0.70 (0.33–1.50)	0.366	0.70 (0.37–1.33)	0.273
60s cohort								
APOE*ε*4*+	1.13 (1.02–1.24)	0.014	1.32 (0.93–1.86)	0.118	1.56 (0.75–3.22)	0.233	1.32 (0.72–2.44)	0.374

IRR, incidence rate ratio; OR, odds ratio; GDS, Goldberg Depression Scale.

### Incidental 4-, 8- and 12-year risk of APOE*ε4*+* for depression

Analysis of the 4-, 8- and 12-year risk of APOE*ε4*+* for depression generally conformed with the earlier analyses over the study period. There was no consistent evidence of APOE*ε4 *+* 4-year risk for any depression (OR = 0.92 (95% CI 0.73–1.15), *P* = 0.433), major depression (OR = 0.73 (95% CI 0.41–1.30), *P* = 0.283), minor depression (OR = 0.95 (95% CI 0.62–1.45), *P* = 0.805) or depressive symptomology according to the GDS (IRR = 1.04 (95% CI 0.98–1.10), *P* = 0.099). There was no 8-year risk of APOE*ε4*+* for any depression (OR = 0.86 (95% CI 0.67–1.10), *P* = 0.221), major depression (OR = 0.96 (95% CI 0.56–1.65), *P* = 0.890), minor depression (OR = 0.74 (95% CI 0.46–1.19), *P* = 0.218) or depressive symptomology according to the GDS (IRR = 1.01 (95% CI 0.96–1.06), *P* = 0.808). There was no 12-year risk of APOE*ε4*+* for any depression (OR = 1.08 (95% CI 0.85–1.36), *P* = 0.533), major depression (OR = 1.30 (95% CI 0.75–2.25), *P* = 0.344), minor depression (OR = 1.17 (95% CI 0.75–1.81), *P* = 0.485) or depressive symptomology according to the GDS (IRR = 1.02 (95% CI 0.96–1.08), *P* = 0.523).

The lack of risk for depression associated with APOE*ε4*+* was generally consistent for all three age cohorts (Table 3). For the 40s cohort, there was marginal evidence for APOE*ε4*+* conferring protection for 4-year risk for any depression status (OR = 0.65 (95% CI 0.41–1.01), *P* = 0.056) and 8-year risk for depressive symptomology (IRR = 0.92 (95% CI 0.84–1.01), *P* = 0.066). For the 60s cohort, there was marginal evidence that APOE*ε4*+* status conferred 4- and 8-year risk increases in depressive symptomology and a 12-year risk of reporting any depression. Overall, lack of consistency in point estimates and the magnitude of the effect sizes suggests no evidence for the role of APOE*ε4*+* in depression. No substantive interactions between APOE*ε4 with gender, years of education and physical health for the whole sample and by age cohort at 4-, 8- and 12-year follow-up were reported.

**Table 3:** The 4-, 8- and 12-year risk of apolipoprotein E ε4 allele (APOE*ε*4*)+ for incident depression

	GDS		Any depression		Major depression		Minor depression	
	IRR (95% CI)	*P*	OR (95% CI)	*P*	OR (95% CI)	*P*	OR (95% CI)	*P*
20s cohort								
APOE*ε*4* + 4-year risk	1.09 (1.00–1.18)	0.042	0.98 (0.69–1.37)	0.881	0.81 (0.36–1.83)	0.619	1.11 (0.62–2.03)	0.716
APOE*ε*4* + 8-year risk	0.99 (0.91–1.08)	0.798	0.80 (0.54–1.18)	0.252	0.88 (0.41–1.88)	0.740	0.81 (0.39–1.66)	0.560
APOE*ε*4* + 12-year risk	0.98 (0.88–1.09)	0.720	0.86 (0.56–1.32)	0.502	1.28 (0.54–3.01)	0.576	0.60 (0.24–1.50)	0.278
40s cohort								
APOE*ε*4* + 4-year risk	0.94 (0.86–1.03)	0.169	0.65 (0.41–1.01)	0.056	0.52 (0.18–1.55)	0.242	0.77 (0.31–1.92)	0.570
APOE*ε*4* + 8-year risk	0.92 (0.84–1.01)	0.066	0.72 (0.45–1.13)	0.155	0.67 (0.25–1.80)	0.429	0.53 (0.20–1.40)	0.203
APOE*ε*4* + 12-year risk	0.97 (0.89–1.07)	0.601	0.92 (0.61–1.39)	0.695	0.95 (0.39–2.31)	0.905	0.86 (0.34–2.19)	0.758
60s cohort								
APOE*ε*4* + 4-year risk	1.11 (1.01–1.22)	0.027	1.22 (0.78–1.92)	0.386	0.75 (0.21–2.69)	0.663	0.83 (0.36–1.95)	0.675
APOE*ε*4* + 8-year risk	1.14 (1.03–1.25)	0.008	1.18 (0.73–1.90)	0.504	3.22 (0.79–13.19)	0.103	0.83 (0.35–1.94)	0.665
APOE*ε*4* + 12-year risk	1.09 (0.97–1.21)	0.138	1.46 (0.99–2.16)	0.056	1.94 (0.54–6.93)	0.310	1.95 (1.05–3.63)	0.034

GDS, Goldberg Depression Scale; IRR, incidence rate ratio; OR, odds ratio.

### Sensitivity analysis: the risk attributed to number of alleles

Results of sensitivity analyses, where a count of alleles substituted for the APOE*ε4+ carrier status, conformed with the main analyses. There was no evidence that possessing higher numbers of alleles was associated with risk for any depression (χ^2^(2) = 0.55; *P* = 0.760), major depression (χ^2^(2) = 0.16; *P* = 0.921), minor depression (χ^2^(2) = 1.94; *P* = 0.379), nor GDS (χ^2^(2) = 5.35; *P* = 0.069) over the study period. This was consistent between age groups and there were few exceptions to this pattern (Table 4). In the 40s cohort, those with 2 APOE*ε4+ alleles reported lower depression symptoms (IRR = 0.70 (95% CI 0.54–0.91), *P* = 0.009). In contrast there was evidence for a dose effect for the 60s cohort with increasing number of depressive symptoms with increasing number of APOE*ε4+ alleles.

**Table 4:** Relationship between number of apolipoprotein E ε4 allele (APOE*ε*4*)+ alleles and incident depression over the study period

	GDS		Any depression		Major depression		Minor depression	
	IRR (95% CI)	*P*	OR (95% CI)	*P*	OR (95% CI)	*P*	OR (95% CI)	*P*
20s cohort								
1 APOE*ε*4*+ allele	1.06 (0.99–1.13)	0.121	0.90 (0.74–1.11)	0.333	1.05 (0.69–1.60)	0.832	0.80 (0.54–1.20)	0.284
2 APOE*ε*4*+ alleles	0.90 (0.72–1.12)	0.348	0.86 (0.46–1.59)	0.630	0.42 (0.06–3.05)	0.389	0.54 (0.13–2.23)	0.394
40s cohort								
1 APOE*ε*4*+ allele	0.95 (0880–1.02)	0.139	0.89 (0.72–1.09)	0.268	0.85 (0.54–1.35)	0.493	0.97 (0.65–1.44)	0.862
2 APOE*ε*4*+ alleles	0.70 (0.54–0.91)	0.009	0.59 (0.21–1.64)	0.311	–^a^		0.57 (0.08–4.19)	0.584
60s cohort								
1 APOE*ε*4*+ allele	1.09 (1.02–1.17)	0.011	1.30 (1.04–1.62)	0.020	1.25 (0.63–2.48)	0.514	1.18 (0.82–1.69)	0.383
2 APOE*ε*4*+ alleles	1.20 (1.01–1.42)	0.039	1.02 (0.48–2.18)	0.958	2.86 (0.66–12.38)	0.159	0.40 (0.05–2.90)	0.363

GDS, Goldberg Depression Scale; IRR, incidence rate ratio; OR, odds ratio.

a. None reported.

The pattern of these results was similar for any-depression risk at 4 years (χ^2^ (2) = 0.52; *P* = 0.771), 8 years (χ^2^(2) = 0.67; *P* = 0.714) or 12 years (χ^2^(2) = 0.46; *P* = 0.796); major depression at 4 years (χ^2^(2) = 1.00; *P* = 0.605), 8 years (χ^2^(2) = 1.19; *P* = 0.552) or 12 years (χ^2^(2) = 1.88; *P* = 0.392), minor depression at 4 years (χ^2^(2) = 0.50; *P* = 0.780), 8 years (χ^2^(2) = 0.54; *P* = 0.764) or 12 years (χ^2^(2) = 1.82; *P* = 0.402), nor GDS at 4 years (χ^2^(2) = 4.85; *P* = 0.089), 8 years (χ^2^(2) = 1.34; *P* = 0.513), or 12 years (χ^2^(2) = 1.50; *P* = 0.473).

These findings were generally consistent in age-stratified analyses; there were few risks identified and these were not consistent (Table 5). For example, in the 20s cohort, those with 1 APOE*ε4+ allele reported increased depressive symptoms at 4 years only (IRR = 1.14 (95% CI 1.02–1.27), *P* = 0.018). Similarly, in the 60s cohort, those with 1 APOE*ε4+ allele reported increased depressive symptoms at 4 years only (IRR = 1.12 (95% CI 1.01–1.25), *P* = 0.039) whereas those with 2 APOE*ε4+ alleles reported increased depressive symptoms at 8 years only (IRR = 1.33 (95% CI 1.02–1.70), *P* = 0.026). Overall, we can conclude no consistent evidence for risk of depression and any risks reported were of a marginal effect size only.

**Table 5:** The 4-, 8- and 12-year risk of number of *A*apolipoprotein E ε4 allele (APOE*ε*4*)+ alleles for incident depression

	GDS		Any depression		Major depression		Minor depression	
	IRR (95% CI)	*P*	OR (95% CI)	*P*	OR (95% CI)	*P*	OR (95% CI)	*P*
*20s cohort*								
4-year risk								
1 APOE*ε*4*+ allele	1.14 (1.02–1.27)	0.018	1.08 (0.78–1.48)	0.658	1.16 (0.57–2.34)	0.673	1.01 (0.55–1.86)	0.972
2 APOE*ε*4*+ alleles	0.94 (0.70–1.27)	0.692	0.92 (0.35–2.40)	0.859	–^a^		0.73 (0.10–5.50)	0.758
8-year risk								
1 APOE*ε*4*+ allele	1.01 (0.93–1.09)	0.841	0.86 (0.59–1.23)	0.402	0.97 (0.48–1.95)	0.924	0.82 (0.41–1.64)	0.583
2 APOE*ε*4*+ allele*s*	0.89 (0.69–1.15)	0.370	0.64 (0.19–2.13)	0.466	–^a^		–^a^	
12-year risk								
1 APOE*ε*4*+ allele	1.00 (0.87–1.15)	0.998	0.86 (0.57–1.30)	0.475	1.23 (0.52–2.90)	0.631	0.58 (0.23–1.45)	0.243
2 APOE*ε*4*+ alleles	0.86 (0.53–1.41)	0.554	1.11 (0.37–3.33)	0.858	1.78 (0.09–14.22)	0.588	1.01 (0.13–7.95)	0.992
*40s cohort*								
4-year risk								
1 APOE*ε*4*+ allele	0.98 (0.92–1.04)	0.481	0.73 (0.49–1.07)	0.102	0.83 (0.36–1.90)	0.665	0.83 (0.38–1.81)	0.644
2 APOE*ε*4*+ alleles	1.10 (0.90–1.34)	0.349	0.82 (0.19–3.51)	0.784	–^a^		1.61 (0.21–12.41)	0.649
8-year risk								
1 APOE*ε*4*+ allele	1.00 (0.94–1.06)	0.980	0.85 (0.58–1.26)	0.432	0.67 (0.27–1.68)	0.395	0.83 (0.40–1.73)	0.620
2 APOE*ε*4*+ alleles	0.84 (0.66–1.06)	0.151	–^a^		–^a^		–^a^	
12-year risk								
1 APOE*ε*4*+ allele	0.98 (0.86–1.11)	0.722	0.93 (0.64–1.36)	0.710	0.87 (0.38–2.00)	0.739	1.28 (0.60–2.71)	0.524
2 APOE*ε*4*+ alleles	0.82 (0.54–1.25)	0.360	1.13 (0.26–4.98)	0.870	–^a^		–^a^	
*60s cohort*								
4-year risk								
1 APOE*ε*4*+ allele	1.12 (1.01–1.25)	0.039	1.22 (0.80–1.88)	0.357	1.16 (0.40–3.32)	0.783	0.80 (0.36–1.79)	0.595
2 APOE*ε*4*+ alleles	0.88 (0.67–1.15)	0.349	0.38 (0.05–2.93)	0.356	–^a^		–^a^	
8-year risk								
1 APOE*ε*4*+ allele	1.09 (1.03–1.21)	0.136	1.21 (0.76–1.92)	0.420	2.33 (0.51–10.62)	0.275	0.83 (0.37–1.87)	0.655
2 APOE*ε*4*+ alleles	1.33 (1.03–1.70)	0.026	2.25 (0.72–7.00)	0.161	9.35 (0.91–96.04)	0.060	1.71 (0.22–13.49)	0.611
12-year risk								
1 APOE*ε*4*+ allele	1.04 (0.92–1.18)	0.535	1.47 (1.02–2.13)	0.040	1.07 (0.27–4.18)	0.927	1.75 (0.95–3.24)	0.075
2 APOE*ε*4*+ alleles	1.36 (0.91–2.06)	0.137	1.08 (0.23–5.00)	0.921	9.22 (0.95–87.30)	0.055	–^a^	

GDS, Goldberg Depression Scale; IRR, incidence rate ratio; OR, odds ratio.

a. None reported.

## Discussion

### Main findings

This study sought to extend current findings^17^ relating to the role of APOE*ε4 as a risk factor for depression by examining the risk over a longer follow-up period and across the adult lifespan. The current study found no risk for incident depression associated with APOE*ε4 at either 4-, 8- or 12-year follow-up in a sample of cognitively intact adults. These findings support another longitudinal study of 633 participants,^25^ however, our study comprises a much larger sample and importantly, examines the risk across the adult lifespan over a longer study period with multiple follow-up observations. Considering the multiple analyses undertaken with multiple forms of the exposure variable (for example APOE*ε4 carrier, number of *ε*4 alleles) and multiple outcomes measures (for example symptomology, any depression, minor or major depression), we conclude that there is no systematic evidence for the role APOE*ε4 in depression risk across the adult lifespan.

### Interpretation of our findings

We note that for the 60s cohort, there was a tendency to report increased depressive symptomology. However, this risk was reported only at the 4- and 8-year follow-up which is in line with previous findings on ‘symptomology’^17^ but this result was not of a substantive magnitude. Given the average number of symptoms between age groups varied from 1.7 to 2.9 symptoms, the IRRs of 1.11 and 1.14 do not reflect a substantive increase that would reflect clinical significance. Further review of these patterns would be needed to determine the extent to which this effect is a consequence of sample power or a phenomenon of importance. If a ‘real’ effect, then questions as to why this risk did not carry through to the 12-year follow-up need to be resolved. In reviewing the data to try and identify possible mechanisms for this pattern, we identified that those in the 60s cohort who reported any depression (OR = 1.53 (95% CI 1.05–2.23), *P* = 0.025) and GDS (IRR = 1.13 (95% CI 1.0–1.20), *P* < 0.001) in the second last wave were more likely not to return in the final wave. These findings suggest further consideration is needed to discriminate risk between acute and chronic depression. However, since our focus is primarily on incident depression, we would emphasise that overall, APOE*ε4 carrier status is unrelated to incident depression. Also, any ‘significant’ GDS effects for the 60s cohort at 4- and 8-year follow-up, and for the 20s cohort at 4-year follow-up, are small effects and would be attenuated when considering other known risk factors for poor mental health. Clearly more work in this area is needed to substantiate those findings reported here and by others.^17,25^

We would also emphasise that we excluded those with GDS score >4 at baseline from the analyses, and although there was a general increase in mean GDS in the 60s cohort from baseline (mean 1.19, s.d. = 1.22) to 12-year follow-up (mean 1.38; s.d. = 1.61), only *n* = 101, *n*=90 and *n*=75 reported GDS scores >4 at 4-, 8- and 12-year follow-up, respectively. So, although there is an increase in GDS scores for the 60s cohort over the 12 years, most are still reporting levels well below a level that might indicate serious psychopathology.

### Comparison with findings from other studies

There are strengths to the current study that contrast with the findings of those studies previously reported.^11,13–15,17–19,25^ The current findings examined multiple observations within individuals over a 12-year period. Many longitudinal studies examine risk of incident depression at a single follow-up, which limits the capacity to capture sufficient incident cases.^14,18^ Further the availability of large longitudinal studies are limited.^14,18^ A further important feature of the current study was that the associations between APOE*ε4 and depression were consistent across the lifespan. It may be that the identified prospective long-term risk previously identified between APOE*ε4 and depression^17^ may be due in part to a sampling of much older participants. It has been suggested that the association between APOE*ε4 and depression is stronger among the very old and those living with dementia or cognitive impairment.^3,11,19^ In contrast participants in the older cohort in the current paper were only 60–64 at baseline and cognitively intact. This might indicate that APOE*ε4 is only associated with depression in the very old and where there is more time for exposure to micro-bleeds and other vascular neuropathology, and for Alzheimer pathology to develop in the critical parts of the cortex.

There is a somewhat paradoxical finding that late-life depression seems to be associated with increased dementia risk but that this may not be the case for mid-life depression. However, a recent meta-analysis of dementia and risk for dementia identified few studies that measured depression at mid-life and therefore part of the lack of early adulthood findings might be at least partly because of the lack of studies with very long follow-up.^37^ Of course, an alternate explanation is that late-life depression develops as part of dementia disease process and not as a precursor or risk factor. If this were the case then one would expect that APOE*ε4 would also predict late-life depression but not early- to mid-adulthood depression. In addition, future genome-wide analysis of gene–gene interactions may indicate that the role of APOE*ε4 in the aetiology of dementia, depression and other disease^1–3,9,10^ is moderated by other genes. This is somewhat supported by inconsistent findings in the risk attributed to APOE*ε4 that could be ascribed to other modifier genes.^11–17^

### Limitations

We recognise that there are limitations that should moderate our findings although these limitations are consistent with other studies.^14–18,25^ Despite using validated measures of depression^30–32^ that have been specifically validated in the PATH sample,^29^ these measures are derived from self-report. Also, we recognise that given the research design in which participants were interviewed every 4 years, we cannot discount the likelihood that participants may develop and be treated for incident depression in the intervening years between our 4-year survey observations that are not captured in our measures of recent (past month) mental health state.

Importantly, however, we have adjusted our analyses to control for the age-related differences in physical health and other comorbidities including education and gender that are likely to have confounded associations between APOE*ε4 status and depression. Whereas relatively small cohort differences were identified for physical health, gender and education, these differences are unlikely to have influenced the current results or their interpretation since we adjusted our analyses to control for physical health, education and gender, which may otherwise have confounded associations between APOE*ε4 status and depression. Recognising that there are normative age-related differences in key mental health risk factors including gender and physical health, as well as age-related differences in mental health itself, it was important to stratify our analyses by age group.

### Implications

In conclusion, this is one of the first population-based study that examined the prospective risk of APOE*ε4 for incidental depression and depressive symptomology at 4-, 8- and 12-year follow-up across the lifespan. Overall, there is little evidence for the APOE*ε4 risk for depression among those who are cognitively intact. We conclude that there is no increased risk for incident depression in adult APOE*ε4 carriers across the lifespan, including among older adults who remain cognitively intact.

## Data Availability

Authors have ongoing access to de-identified/re-identifiable study data. The lead author has the data and syntax specifically used for the analysis of the current paper. Further information about the data can be found at: https://www.pathstudy.org.au/
